# The Influence of Sex Steroids on Structural Brain Maturation in Adolescence

**DOI:** 10.1371/journal.pone.0083929

**Published:** 2014-01-08

**Authors:** P. Cédric M. P. Koolschijn, Jiska S. Peper, Eveline A. Crone

**Affiliations:** 1 Institute of Psychology, Brain and Development Lab, Leiden University, Leiden, The Netherlands; 2 Leiden Institute for Brain and Cognition, Leiden, The Netherlands; 3 Brain and Cognition, University of Amsterdam, Amsterdam, The Netherlands; 4 Department of Developmental Psychology, University of Amsterdam, Amsterdam, The Netherlands; Centre Hospitalier Universitaire Vaudois Lausanne - CHUV, UNIL, Switzerland

## Abstract

Puberty reflects a period of hormonal changes, physical maturation and structural brain reorganization. However, little attention has been paid to what extent sex steroids and pituitary hormones are associated with the refinement of brain maturation across adolescent development. Here we used high-resolution structural MRI scans from 215 typically developing individuals between ages 8–25, to examine the association between cortical thickness, surface area and (sub)cortical brain volumes with luteinizing hormone, testosterone and estradiol, and pubertal stage based on self-reports. Our results indicate sex-specific differences in testosterone related influences on gray matter volumes of the anterior cingulate cortex after controlling for age effects. No significant associations between subcortical structures and sex hormones were found. Pubertal stage was not a stronger predictor than chronological age for brain anatomical differences. Our findings indicate that sex steroids are associated with cerebral gray matter morphology in a sex specific manner. These hormonal and morphological differences may explain in part differences in brain development between boys and girls.

## Introduction

Adolescence is a highly important transition phase between childhood and adulthood, marked by significant physical, social, cognitive and emotional changes [1]. Puberty is roughly characterized as the onset of adolescence between ages 9–14 (approximately 1–2 years earlier in girls than in boys) and represents the period in which reproductive capacity is achieved and phenotypic sexual maturity attained [2]. Puberty starts with pulsatile secretion of luteinizing hormone (LH) and the gonadotropin follicle-stimulating hormone (FSH). These hormones stimulate gonadal growth and gonadal hormone secretion and increase more than 30-fold in boys and 100-fold in girls [3]. This major hormonal change occurs as a result of activation of the hypothalamic-pituitary-gonadal (HPG) axis, causing gonadal maturation and production of sex steroids, most notably testosterone in boys and estradiol in girls. This process is termed gonadarche, and is followed by the activation of the growth axis leading to well known growth spurts and changes in body composition in puberty [1], [4], [5].

Besides clearly visible physical changes, puberty also reflects a period of structural brain reorganization and pruning of neuronal circuits in the brain [6], [7]. Sex steroids are thought to play an additional role in the refinement of brain maturation during puberty [8].

While it is known that these hormonal increases are associated with a cascade of temperamental and behavioral changes (more risk-taking, social and exploratory behavior) [9], the relationship between the levels of circulating gonadal hormones and brain morphology is not yet well understood.

Only recently studies started to examine the association between circulating sex steroids and brain gray matter in adolescence, with the majority of studies focusing on testosterone. Associations between testosterone levels and brain maturation vary widely between brain regions, morphological parameters (i.e. volume, cortical thickness, surface area) and sex. High circulating testosterone levels have been associated with smaller hippocampal and larger amygdala volumes [10], larger amygdala volumes in females only [11], and larger hypothalamic volumes in boys [10]. Studies reporting on the association of cortical thickness and testosterone demonstrated thicker limbic and occipital cortices with higher levels of testosterone in boys, but the opposite in girls [12]. Finally, in a longitudinal study higher levels of testosterone were related to thinning in the left dorsolateral prefrontal cortex, and primarily in portions of the left cingulate cortex in boys, whereas in girls there was a positive to negative association with testosterone in the right somatosensory cortex [13]. Thus, the findings on the relation between brain development and testosterone show inconclusive evidence and differ depending on sample size and age-range to detect differences.

With respect to estradiol, two studies found higher levels in early pubertal girls to be associated with a larger parahippocampus and uncus volumes [10], greater gray matter density in middle frontal-, inferior temporal-, and middle occipital gyri, but lower regional gray matter density in decreases within prefrontal, parietal and middle temporal areas [14]. Thus, the relation with estradiol is much less studied and the differences reported are based on relatively small samples with small age-ranges.

While these studies do not show converging evidence of the direction of how sex steroids influence maturation of brain structures, it is clear that there are sex-specific associations between sex hormones and (sub)cortical brain areas which may vary over time. Next to sex steroids, the pituitary hormone LH -the hormonal precursor to the production of sex steroids- has also been implicated in brain maturation in very early stages of puberty [15]. Indeed, from both animal and human research it is known that LH receptors are found throughout the brain, including the parietal cortex [16], [17]. However, it is still unknown if and how circulating LH levels exert their effects on cerebral gray matter across a wider age-range.

The aim of the current study was to assess the association between testosterone, estradiol, LH and (sub)cortical brain volumes, cortical thickness and surface area in a large Dutch sample (N = 215). We also selected specific regions of interest based on their relevance in functional brain maturation studies in adolescence [18], including the anterior cingulate cortex (ACC), dorsolateral prefrontal cortex (DLPFC), the inferior frontal gyrus (IFG) and orbitofrontal cortex (OFC). For example, fMRI studies of executive function have demonstrated increased recruitment of DLPFC and ACC from childhood to adulthood [19]. In addition, increased activity of OFC in adults relative to children and adolescents has been associated with a more matured pattern of self-regulatory processes [20].

In a seminal study by Raznahan et al. [21] stepwise effects of androgen receptor transcriptional activity across adolescence were associated with cortical thinning in the inferior frontal gyrus (IFG), a region critically involved in executive functions such as inhibition. Specifically, girls with high androgen function demonstrated a significantly thinner IFG volume at age 22 years than at age 9 years compared to girls with low function or boys.

Evidence from genetic endocrine disorders such as familial male precocious puberty (a rare disorder with selective androgen excess) and Klinefelter syndrome (androgen insufficiency, in which males possess one extra X-chromosome) suggests an association between thinner DLPFC and high and low levels of testosterone respectively during adolescence [22]. In females with Turner syndrome (a genetic disorder characterized by partial or complete absence of one of the two X chromosomes), aberrant activation patterns have been reported for a.o. DLPFC [23]. Finally, in young adults, circulating levels of estradiol have been associated with thinning of the inferior frontal gyrus irrespective of sex [24]. We hypothesized that increasing levels of testosterone and estradiol would primarily be related with maturation (i.e. thinning or smaller volumes) of these frontal brain regions [12], [13].

Finally, we also explored associations of pubertal stage, as assessed by self-report with the Pubertal Development Scale (PDS; [25]), and brain anatomy, to explore whether pubertal stage is a stronger predictor than chronological age.

## Methods

### Participants

A total of 215 right-handed participants between ages 8–25 were recruited from local schools and advertisement and included in this study. All participants had normal intelligence (M 109.31, SD 10.78; approximated using block design and similarities of the WISC-III for children up to 16 years of age and of the WAIS-IV from 16 years and older; **Table 1**) Participants had no self-reported history of neurological or psychiatric disorders, chronic illness, learning disabilities, or use of medicines known to affect nervous system functioning. Participants and primary caregivers (for minors) gave written informed consent for the study. Adults received fixed payment for participation, whereas children and their parents received presents and travel reimbursement. The internal review board from the Leiden University Medical Center approved the study.

**Table 1:** Demographic variables.

	Description	N	Mean	SD
**Female**	**Age**	112	14.29	3.78
	** range**		[8.41–23.99]	
	**IQ**	111	108.60	10.26
	** range**		[85–130]	
	**PDS**	105	3.29	1.31
	** pre**	13		
	** early**	13		
	** mid**	36		
	** late**	17		
	** post**	26		
	Testosterone (pmol/l)**	101	19.25	13.77
	** range**		[4–83]	
	Estradiol (pmol/l)**	102	0.91	0.44
	** range**		[0.16–2.18]	
	**LH** a (U/l)**	90	1.76	2.18
	** range**		[.10–14.6]	
	LH-Creatinine*	90	0.14	0.15
	** range**		[0.01–0.89]	
**Male**	**Age**	103	14.55	3.84
	** range**		[8.01–25.96]	
	**IQ**	98	110.07	11.31
	** range**		[80–137.5]	
	**PDS**	101	2.97	1.4
	** Pre**	21		
	** early**	18		
	** mid**	24		
	** Late**	19		
	** Post**	19		
	Testosterone (pmol/l)**	91	225.55	200.04
	** range**		[4–915]	
	Estradiol (pmol/l)**	94	0.93	0.46
	** range**		[0.30–3.01]	
	**LH** a (U/l)**	90	3.32	2.52
	** range**		[0.20–15.20]	
	LH- Creatinine*	90	0.19	0.11
	** range**		[0.02–0.50]	
**Total**	**Age**	215	14.41	3.81
	** range**		[8.01–25.96]	
	**IQ**	214	109.31	10.78
	** range**		[80–137.5]	
	**PDS**	206		
	** pre**	34		
	** early**	31		
	** mid**	60		
	** late**	36		
	** post**	45		
	**Testosterone (pmol/l)**	192	117.03	172.11
	** range**		[4–915]	
	**Estradiol (pmol/l)**	196	0.92	0.46
	** range**		[.16–3.01]	
	**LH** a **(U/l)**	180	2.54	2.48
	** range**		[0.1–15.20]	
	**LH- Creatinine**	180	0.166	0.13
	** range**		[0.01–0.89]	

^a^ 19 Females and 14 Males below detection limit, not determined in 9 females.

Significant differences between males and females: *p<.05; **p<.005.

Abbreviations: PDS, Puberty Developmental Scale.

### Pubertal Measures

#### Puberty Developmental Scale (PDS)

The Pubertal Development Scale [25] was completed by all participants up to the 18 years of age. At a 4-points scale, participants had to indicate whether secondary sexual characteristics had: 1) not yet started to develop, 2) were showing the first signs, 3) were showing very clear development or 4) had already finished developing [26]. Puberty Category Scores were subsequently calculated and were based on body hair growth, voice change and facial hair growth for boys, and body hair growth, breast development and menarche for girls, leading to five categories: pre-pubertal, early pubertal, mid-pubertal, late pubertal and post-pubertal (Table 1).

#### Sex steroids

Boys and girls collected their saliva and urine at home, directly after waking up. Participants were instructed not to eat or brush their teeth before collecting saliva. It is most desirable to enroll girls during a specific period during the menstrual cycle to control for hormonal fluctuations, but most neuroimaging studies rarely take menstrual phase into account due to practical considerations. Here post-menarcheal girls collected saliva samples on the same day within the early follicular phase of the menstrual cycle (day 7), when hormone levels (e.g., progesterone) are relatively low [4], [27]. Similarly, girls using oral contraceptives (n = 6) collected a saliva sample on the last day within their stopping period (day 7). Girls using contraceptives without a stopping period, such as hormonal intrauterine devices (e.g. Mirena), were excluded from participating in this study. All results remained similar with exclusion of girls using contraceptives; therefore, all results reported here include the whole sample.

The saliva samples of boys and girls were assayed for testosterone and estradiol levels at the Department of Clinical Chemistry of the VU Medical Center. The lower limit of detection was 4 pmol/L for testosterone, and 0.1 pg/ml for estradiol. Salivary testosterone was determined by isotope dilution–online solid phase extraction liquid chromatography–tandem mass spectrometry [28]. Intra-assay coefficient of variation (CV) was 11% and 4%, at 10 and 140 pmol/L, respectively and inter-assay CV was 8% and 5%, at 31 and 195 pmol/L, respectively. Salivary estradiol was determined using an enzyme linked immunosorbent assay (ELISA; DRG-Instruments, Marburg, Germany). Inter-assay CV was 8% and 15% at 10 and 40 pg/L, respectively [29]. LH was determined in morning urine using highly sensitive immunometric assays (Luminiscention) detection limit 0.1 U/l, carried out by the endocrinological laboratory of Clinical Chemistry of the VU Medical Center in Amsterdam (Architect, Abbott Laboratories, Abbott Park, Illinois USA). Urinary LH levels were divided by creatinine level to correct for variations in urine excretion rate [15]. A creatinine correction has been demonstrated to enhance the detection of LH-surges [30]. Hormonal levels for each sex are displayed in **Table 1**.

### Data Acquisition

All participants were scanned on a 3-Tesla whole body Philips Achieva MRI system (Best, The Netherlands). High-resolution T1-weighted anatomical scan were obtained: 3D-T1-weighted scan: TR = 9.717 msec; TE = 4.59 msec, flip angle = 8°, 140 slices, .875×.875×1.2 mm, FOV = 224.000×168.000×177.333). All anatomical scans were reviewed and cleared by a radiologist.

### Image Analysis

Cortical reconstruction and volumetric segmentation was measured automatically using FreeSurfer5.0 (http://surfer.nmr.mgh.harvard.edu/, [31], [32]. Details of the surface-based cortical reconstruction and subcortical volumetric segmentation procedures have been extensively documented previously [31]–[34]. Briefly, the FreeSurfer pipeline performs motion correction on the T1-images, automatically removes non-brain tissues [34], transforms volumetric data to a common atlas, performs intensity normalization and topology correction [33], [35] and defines the boundaries of the gray/white matter and pial surface [31], [32]. Volumetric subcortical segmentation and measurement was performed using automated procedures that have been validated as comparable in accuracy to much slower, labor-intensive manual tracing and labeling methods [33], [36]. This procedure automatically classifies brain tissue into multiple distinct structures such as cerebral and cerebellar gray and white matter, cerebrospinal fluid (CSF), basal ganglia, and other subcortical structures. Using probabilistic information derived from a manually labeled training data set, this approach automatically assigns a neuroanatomical label to each voxel in the MRI volume. For the purposes of the current study, automated image surfaces and segmentations were inspected and screened for quality control but were not manually edited, in order to maintain the objectivity of results. Of note, large deformities such as failure to segment or include the entire brain were excluded from the study (N = 20, not included in **Table 1**). Intracranial volume was determined by a validated automated method known to be equivalent to manual intracranial volume estimation [37].

Regions of interest (ROIs) were created based on their relevance in functional brain maturation in adolescence [18]. These regions were created based on combined labels from the Desikan–Killiany atlas [38]: ACC (rostral, caudal, posterior and isthmus parcellation); OFC (lateral and medial OFC parcellation); inferior frontal gyrus (IFG; pars opercularis, pars orbitalis and pars triangularis), DLPFC (middle frontal gyrus, inferior and middle frontal sulci). Gray matter volume (ml) and cortical thickness (mm) were automatically extracted.

### Statistical Analyses

Testosterone and LH/creatinine levels were highly skewed and were log-transformed. All further analyses concerning levels of LH/creatinine were carried out on these log-transformed scores. As expected, there were substantial sex differences in testosterone levels (*F* = 125.6; *p*<.00001), therefore a Z-transformation was applied on the log-transformed testosterone levels: high scores on this testosterone distribution indicated high levels of testosterone relative to other individuals of the same sex [39], [40]. All further analyses concerning levels of testosterone were carried out on these log-transformed, standardized scores of testosterone.

Age was positively associated with testosterone (whole sample: r = .62; p<.001; Males: r = .72, p<.001; Females: r = .53, p<.001), estradiol (whole sample: r = .23, p = .001; Males: r = .32, p = .002; Females: r = .13, p = .19), and LH (whole sample: r = .22; p = .003; Males: r = .18, p = .1; Females: r = .29, p = .018). To examine the inter-relations between sex steroids, partial correlations were calculated between hormone levels correcting for age. In males, testosterone levels showed a moderate association with estradiol levels (r = .23; p = .036), and with LH (r = .31; p = .005), but no association was found between estradiol and LH levels (r = .07; p = .51). In females, testosterone levels showed a moderate association with estradiol levels (r = .27; p = .009), and with LH (r = .36; p = .001), and also between estradiol and LH levels (r = .24; p = .025).

The cortical thickness data were averaged across participants in the spherical coordinate system after smoothing (FWHM 10 mm), so that surface areas with significant differences of mean cortical thickness differences and the different sex steroids levels could be overlaid in statistical difference maps (using *t*-statistics) for the whole sample and between boys and girls. Vertex-wise analyses were performed using a general linear model approach in QDEC. We addressed differences in cortical thickness for the whole sample and each hormone, with age as nuisance factor. Differences were reported as significant below a FDR corrected p-value of 0.05.

The volumes of all subcortical structures and ROIs were averaged across hemisphere within participants. Brain volume measures were corrected for intracranial volume as an estimate of head size, because head size in males is in general about 10% larger than in females [41]. Stepwise regression analyses were performed on the whole sample with age, sex, and pubertal hormone (testosterone, estradiol or LH; or a sex-by-hormone interaction-term) as predictors. The analyses were repeated within each sex separately. Due to the number of analyses, we corrected for multiple comparisons using a Bonferroni correction: α = .05/13ROIs = .0038.

To explore if pubertal stage is a stronger predictor than chronological on brain anatomy, we performed whole brain vertex-wise analyses and ROI analyses with pubertal stage (pre, early, mid, late and post) and age as a nuisance variable. In case of significant effects of pubertal stage, we tested the precise differences using Tukey post hoc tests.

## Results

### Whole Brain Vertex-wise Analyses with Sex Steroids

**Figure 1:**
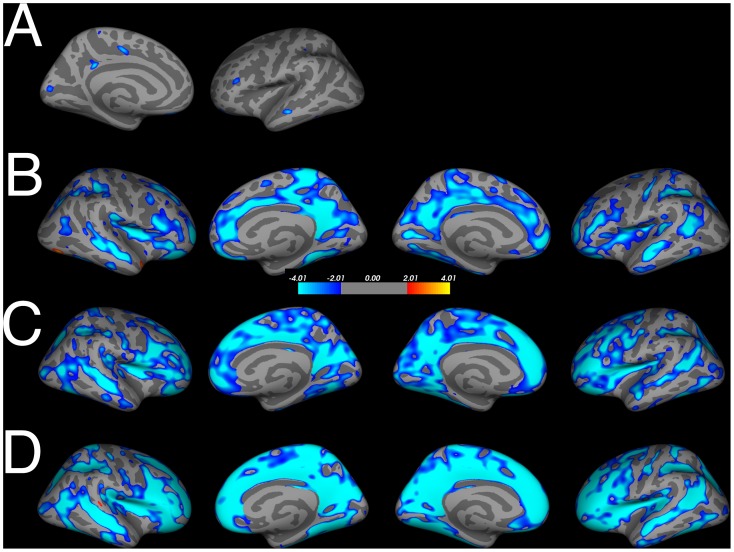
Overlap of testosterone and estradiol levels (corrected for age), age effects corrected for testosterone levels and general age-effects on cortical thickness. A. Estradiol (pmol) related thinning. B. Testosterone (Zlog) related thinning. C. Age-related thinning corrected for testosterone levels (Zlog). D. Age-related thinning. FDR-corrected, p<.05 in all figures.

In contrast with our hypothesis, there were no effects of sex steroids on cortical thickness and surface area (with age as nuisance variable and correction for multiple comparisons). Subsequent analyses with each hormone and age separately (corrected for multiple comparisons), revealed that there was significant overlap in brain regions that showed a correlation with testosterone or estradiol and age, but not LH, suggesting that the age-related difference were stronger than the hormone-related differences (for testosterone and estradiol, see **Figure 1A,B**). Age-related thinning remained similar after correction for sex hormone levels (for testosterone see **Figure 1C,D** **)**. These age-related findings have been reported in several other studies (e.g. [42]–[44]). Next, we used the region-of-interest approach to examine the unique contribution of sex steroids after controlling for age effects.

### Relationship between Brain Volumes and Sex Steroids

#### Testosterone

Testosterone related effects, after controlling for age-related differences, were found in ACC and OFC gray matter volume. Specifically, we found that higher testosterone levels contributed to ACC (p = .008) and OFC (p = .026) gray matter volume maturation, i.e. additional volume reductions after controlling for age-related volume reductions (see Table 2A). The sex-by-testosterone interaction was significant for the ACC after controlling for age (p<.001), but not for OFC (p = .25). To further explore possible sex differences, we repeated the analyses in each sex separately for both structures. These analyses showed that for males higher testosterone levels were related to smaller ACC gray matter volumes (p = .002; survived Bonferroni correction), but in females there was no such association (p>.5). The opposite was found for the OFC, such that higher testosterone levels in females were related to smaller OFC gray matter volumes (p = .023), but not in males (p>.4), but this effect did not survive Bonferroni correction.

**Table 2:** Regression analyses between sex steroids and gray matter volumes.

A. Regression with Age & Testosterone						
Brain area	Group N = 192	Description	ß	F model	R^2^	*p* F-change
Cingulate GM VOL^b^	All	Age	−.490	110.07	.367	**<.0001** a
		TES	−.195	60.08	.391	.008
		Sex	.071	41.15	.396	.216
	Males	Age	−.334	49.66	.358	**<.0001** a
		TES	−.366	32.11	.422	**.002** a
	Females	Age	−.609	68.42	.409	**<.0001** a
		TES	−.056	34.18	.411	.542
OFC GM VOL^c^	All	Age	−.498	99.15	.343	**<.0001** a
		TES	−.157	53.14	.360	.026
		Sex	.139	38.28	.379	.017
	Males	Age	−.508	43.57	.329	**<.0001** a
		TES	−.090	21.92	.333	.476
	Females	Age	−.522	65.86	.399	**<.0001**
		TES	−.207	37.02	.430	.023
**B. Regression with Age & Estradiol**						
**Brain area**	**Group N = 196**	**Description**	**ß**	**F model**	**R^2^**	***p*** ** F-change**
Total GM VOL^c^	All	Age	−.708	212.71	.523	**<.0001** a
		Es	−.098	109.65	.532	.057
		Sex	.176	82.40	.563	**<.0001** a
	Males	Age	−.639	87.73	.488	**<.0001** a
		Es	−.185	49.02	.519	.018
	Females	Age	−.770	149.28	.599	**<.0001** a
		Es	−.032	74.21	.600	.616
						
Cingulate GM VOL^c^	All	Age	−.571	116.24	.375	**<.0001** a
		Es	−.192	66.93	.410	**.001** a
		Sex	.055	44.94	.413	.325
	Males	Age	−.518	49.51	.350	**<.0001** a
		Es	−.227	29.84	.396	.01
	Females	Age	−.631	73.58	.424	**<.0001** a
		Es	−.150	39.87	.446	.049
**C. Regression with Age & LH**						
**Brain area**	**Group N = 180**	**Description**	**ß**	**F model**	**R^2^**	***p*** ** F-change**
Total GM VOL^b^	All	Age	−.722	186.35	.511	**<.0001** a
		LH	−.014	93.64	.514	.327
		Sex	.194	71.00	.548	**<.0001** a
	Males	Age	−.726	85.12	.486	**<.0001** a
		LH	.144	45.59	.512	**.**062
	Females	Age	−.736	124.78	.586	**<.0001** a
		LH	−.122	65.35	.591	.085

^a^ Survives Bonferroni correction (α = .0038).

^b^ significant sex-by-hormone interaction.

^c^ non-significant sex-by-hormone interaction.

Testosterone levels are defined as the Z-transformation of the log-transformed testosterone levels; Estradiol levels are in pmol/l; LH levels were divided by creatinine levels to correct for variations in urine excretion rate, and log-transformed. All volumes were larger in males compared to females.

Abbreviations: ES, Estradiol; GM, Gray matter; OFC, Orbitofrontal cortex; TES, testosterone; VOL, volume.

### Estradiol

A significant effect of estradiol levels was found also in ACC gray matter volume, demonstrating smaller ACC gray matter volumes with higher estradiol levels after controlling for age-related effects (p = .01; see **Table 2B**). The sex-by-estradiol interaction was not significant for the ACC after controlling for age (p = .35). Yet, to further explore possible sex differences, we repeated the analyses in each sex separately. These analyses showed that higher estradiol levels were associated with smaller ACC gray matter volume in males (p = .01), and in females (p = .049).

For total gray matter volume the sex-by-estradiol interaction was not significant (p = .088). Subsequent analyses revealed an association between estradiol and total gray matter volume in males after controlling for age (p = .018). However, none of the estradiol-brain volume associations survived Bonferroni correction.

### LH

We found a significant sex-by-LH interaction with total gray matter volume after controlling for age (p = .02; **Table 2C** **)**. Follow-up analyses in males and females resulted in trend level associations with gray matter (Males: ß = 1.44; p = .062; Females: ß = −.122; p = .085). As the only earlier study on LH and brain structure found effects in early pubertal children [15], we repeated the LH analyses by removing participants in advanced stages of puberty (i.e. mid, late and post puberty). No significant effects of LH on total gray matter were found after controlling for age.

There were no significant associations between DLPFC, IFG or subcortical structures and testosterone, estradiol or LH after controlling for age effects.

### Pubertal Stage

To examine if pubertal stage is a stronger predictor than is chronological on brain anatomy, we performed whole brain vertex-wise analyses and ROI analyses with pubertal stage (pre, early, mid, late and post) with age as a nuisance variable.

There were no effects of pubertal stage based on PDS on cortical thickness and surface area (with age as nuisance variable and correction for multiple comparisons).

We used the GLM to examine the association with pubertal stage and our ROIs, here corrected for both intracranial volume and age, and Tukey post hoc tests to examine possible differences between pubertal stages. No significant effects were found for our subcortical structures or our custom ROIs, indicating that pubertal stage, based on self-report, is not a stronger predictor for brain anatomy than chronological age.

To compare our findings with two earlier studies [11], [45], we repeated our analyses and excluded the pre and post-puberty group, leaving three groups: early (N = 31, 18 Males), mid (N = 60, 24 Males) and late puberty (N = 36, 19 Males). Here we found an association between pubertal stage and inferior frontal gyrus (IFG) gray matter volume (F_(2,137)_ = 3.94; p = .033) independent of age, sex and intracranial volume. Tukey post hoc tests indicated that IFG volume was larger in early pubertal stages compared to late puberty (p = .025), but did not differ with mid puberty (p = .52) or between mid and late pubertal stages (p = .14).

## Discussion

The goal of this study was to examine the contributions of the sex steroids testosterone, estradiol and LH to structural brain maturation in a large Dutch sample with variability in both age and pubertal status. Two main effects were reported: 1) Our results indicate a sex-specific difference in testosterone related influences on gray matter volume of the anterior cingulate after controlling for age effects. Although other associations between sex steroids and brain anatomy were reported, these findings did not surive stringent Bonferroni correction. 2) Pubertal stage based on self-report was not a stronger predictor than chronological age for brain anatomy. The specific findings will be discussed below.

### Testosterone

Circulating testosterone levels were inversely related to gray matter volume in the ACC and OFC. There were sex-specific differences in testosterone-brain associations, similar to the study by Bramen et al (2012) where sex-specific differences were reported in left inferior parietal lobule, middle temporal gyrus, calcarine sulcus, and right lingual gyrus. Here, testosterone levels in males, but not in females, were strongly related to a smaller ACC gray matter volume after controlling for age-related volume reduction. Our effects of testosterone mimic prior findings from a longitudinal developmental neuroimaging study in which ACC cortical thinning was related to higher levels of testosterone in males only [13].

Decreased gray matter volumes during puberty and adolescence have been associated with the loss of unneeded connections (synaptic pruning; [46]), decreases of dendritic spine density and elimination of synaptic spines starting during puberty [47], and the encroachment of continued white matter growth which normally extends into the 4th decade ([48] [49]–[51]. Interestingly, in a recent study cultured nerve cells were exposed to high levels of testosterone, and it was found that it triggered programmed cell death, but not with low or normal levels of testosterone or estradiol [52]. Testosterone is key to the development, differentiation and growth of cells, but it was found that very high levels result in opposite effects. However, it would be too simplistic to suggest that testosterone levels are the only basis for these volumetric sex differences. Currently, the mechanism(s) through which testosterone and estradiol direct neural changes in the brain are largely unknown [53].

On a behavioral level, in numerous studies on animals, a strong correlation between testosterone levels and aggression has been reported (e.g. [54]), while in humans testosterone is linked to dominance and competitiveness, in general, more than to aggression (e.g. [55]). During adolescence, competitiveness and increased risk taking behavior is characteristic of males [56]. In a longitudinal study in adolescent boys, a positive correlation was found between testosterone and different forms of aggressive and delinquent behavior [57]. Furthermore, aggressive and defiant behavior has been associated with smaller ACC volumes [58] and thickness [59] in pubertal boys (ages 6–18). Speculatively, our finding of smaller ACC volumes in males and increased levels of testosterone may predispose to more competitive and dominant behaviors characteristic for adolescence.

In females we found an association between higher testosterone levels and smaller OFC gray matter volume after correcting for age (but not after Bonferroni correction). The OFC has been thought to play a crucial role in optimal decision-making under risk and value-based decision-making [60]. Increased risk-taking behavior during adolescence has been associated with slow development of frontal regions, necessary for top-down control and inhibitory processes, as compared to earlier maturation of subcortical brain structures related with reward and sensation seeking [20], [61]. The relationship between testosterone and OFC is demonstrated by studies measuring endogenous testosterone levels showing reduced OFC involvement during impulse control [62], sex-specific differences in risk-taking behavior and OFC morphology [63] and reduced OFC-amygdala functional coupling in a testosterone administration study. [64] Taken together, these findings may serve as a potential explanation for sex differences in decision-making and risk-taking behavior during adolescence.

### Estradiol

Similar to the effects of testosterone, estradiol showed a negative association with ACC gray matter volume in males and females, though the sex-by-estradiol interaction was not significant. Estradiol levels were inversely related to cerebral gray matter volume in males, but not in females. It should be noted that the enzyme aromatase converts a small portion of testosterone into estradiol [65], which may in part account for our current findings, despite low correlations between these pubertal hormone levels. Although evidence for the relation between estradiol and gray matter development during adolescence is scarce, earlier studies (with smaller sample sizes and different methodologies) reported decreased gray matter density in frontal and parietal regions in females with higher estradiol levels [10], [14]. Thus, the current results show that in the absence of sex-differences in estradiol levels, there is a male-specific effect of estradiol on ACC and cerebral gray matter. However, caution must be taken with interpreting our findings, as estradiol-brain effects did not remain statistical significance when correcting for multiple comparisons.

### Luteinizing Hormone

Here we report a significant sex-by-LH interaction for cerebral gray matter in humans. As can be seen in Table 2C, males seem to show a slight positive association with cerebral gray matter while it is the opposite in females; but none of these findings reached statistical significance. Although LH receptors are distributed throughout the brain, including hippocampus, cortex, area postrema, hypothalamus and cerebellum [16], [17], the effects of LH on brain tissue may be too small to detect differences at a global and local level. The only earlier study on LH and brain structure demonstrated positive effects of LH in early pubertal children on white matter [15]. That study was specifically designed to assess sex steroids in a homogeneous sample of nine-year old twin pairs (N = 104 individuals). Here, our focus was on the interplay of sex steroids and structural brain development across the wider range of adolescence. Nevertheless, we looked into the relationship between LH and brain morphology in pre, early, and mid-puberty, but no evidence for an association between LH and gray matter was found.

### Review of Non-significant Sex-steroid Findings

No significant associations between pubertal hormone levels and hippocampal and amygdalar, or other subcortical brain structures were found. This was unexpected, because the hippocampus and amygdala are both rich in testosterone and estrogens receptors [66] and testosterone administration in females leads to more male-typical behavior [55]. Furthermore, positive associations of testosterone have been reported in hippocampal and amygdalar volumes in puberty [10], [11]. The absent findings between sex steroids and subcortical brain morphology might be explained by a number of factors. First, age-related brain changes may outweigh hormonal effects, especially in samples with a relatively large age-range. Second, we aimed to assess biological variation in estradiol levels, rather than menstrual cycle variation. Hence, estradiol levels in our sample were relatively low in girls, because saliva was collected during the early follicular phase of their menstrual cycle, when hormone levels are low [27]. A few studies in humans have started to look into the effects of menstrual cycle phase and brain morphology. Although, sample sizes are small, there is some evidence of phase-related changes in amygdala and cerebral gray matter volume [67], [68]. This emphasizes the need to be precise in data collection during the menstrual cycle. Third, subcortical structures may mature more pronounced under the influence of fetal testosterone. In a recent study, fetal testosterone levels of boys aged 8–11 predicted differences in gray matter volume in some sexual dimorphic brain structures [69].

Interestingly, functional brain imaging studies reported relations between testosterone levels and brain activation mainly in subcortical areas, such as the ventral striatum [70]. It will be interesting to combine these approaches in future studies, and examine the role of stable versus fluctuating levels of sex steroids on brain structure, function and behavior.

### Pubertal Stage versus Chronological Age

Besides the effects of sex-steroids we also looked into the contribution of pubertal stage as a predictor of brain anatomy compared to chronological age. We did not find any differences between groups on the whole brain analyses, or the ROI approach when all pubertal stages were included. This is in line with Bramen and colleagues, who reported associations in amygdala volume in boys with pubertal stage, but these effects were lost after age correction [11]. In two independent studies, pubertal stage was found to be a better predictor than chronological age in females for the left amygdala [45], and for the right amygdala and bilateral cortex [11]. Here we could not replicate these findings, even when we removed the pre and post-puberty group from the analyses. However, when only early, mid and late pubertal groups were included in the analyses there was an effect of pubertal stage on IFG volume, such that early pubertal groups had larger volumes compared to late pubertal groups independent of age, sex and intracranial volume. This finding complements earlier reports demonstrating smaller IFG volumes in girls with increased estradiol levels [14], and in part Witte et al. showing smaller IFG volumes with high levels of testosterone, but larger volumes for high levels of estradiol in young adults [24].

### Methodological Considerations

One may argue that the age distribution of our sample could hamper detection of some hormone-related differences, especially on a whole brain level. In the current study, age-related effects outweighed hormonal effects (see **Table 2** and **Figure 1**). The advantage of the current study is that we were able to examine sex hormone-related influences across adolescence. However, studies with a narrow age-range may be more suitable to explicitly disentangle sex steroids and age effects. In addition longitudinal designs are needed to assess hormonal and brain changes over time, and confirm reliability of hormonal assessment during puberty.

In the current study, hormones were collected immediately after waking up for several reasons: First, LH shows a day-night rhythm, while testosterone and estradiol show diurnal rhythms. Second, to reduce saliva contamination due to for example food or drinks at a later time point. Third, this method was used to reduce the effort for the participants and parents, and increase compliance. However, during puberty there is a shift in testosterone peak levels, from early morning in pre- and mid-puberty to the afternoon in late puberty [71], [72]. Furthermore, diurnal rhythms of testosterone are more stable in pubertal boys compared to girls [73]. Estradiol shows a similar diurnal pattern in girls, with high levels in the morning and low levels in the evening, but several studies have shown that the diurnal pattern for estradiol diminishes after menarche and is lost between 1–2 years postmenarche [71], [74]. These fluctuations in diurnal patterns may have influenced our results. In future studies it will be important to collect multiple saliva samples (across multiple days) to look at influence of diurnal rhythms and the relation with structural brain development.

## Conclusions

In sum, sex steroids are associated with cerebral gray matter morphology in a sex specific manner. Specifically, our results indicate age independent, sex-specific differences in testosterone related influences on gray matter volumes of the anterior cingulate. The functional relevance of these hormone-related findings to our understanding of typical brain development could be vast numerous. For example, the influence of sex steroids on human brain structure not only gives important insights into the etiology of healthy brain maturation, but can also provide valuable information for the development of neuropsychiatric illnesses with a skewed sex ratio [6], [75]. In future studies, it is important to reach consensus on standardized measures for hormone collection to reduce unnecessary between-study variability. Second, to understand the functional relevance of hormone-related brain maturation, future studies should effectively combine behavioral, functional and hormonal measurements to disentangle the structure–function-hormone relationship and its development during the transition into adulthood.
